# Enhancing Confidence and Interpretability of a CNN-Based Wafer Defect Classification Model Using Temperature Scaling and LIME

**DOI:** 10.3390/mi16091057

**Published:** 2025-09-17

**Authors:** Jieun Lee, Yeonwoo Ju, Junho Lim, Sungmin Hong, Soo-Whang Baek, Jonghwan Lee

**Affiliations:** 1 Department of System Semiconductor Engineering, Sangmyung University, Cheonan 31066, Republic of Korea; 202221364@sangmyung.kr (J.L.); 202221376@sangmyung.kr (Y.J.); 202021320@sangmyung.kr (J.L.); 202021334@sangmyung.kr (S.H.); 2 Department of Human Intelligence and Robot Engineering, Sangmyung University, Cheonan 31066, Republic of Korea

**Keywords:** convolutional neural network, deep learning, semiconductor, wafer map, defect analysis, local interpretable model-agnostic explanations, temperature-scaling, gradient-weighted class activation mapping

## Abstract

Accurate classification of defects in the semiconductor manufacturing process is critical for improving yield and ensuring quality. While previous works have mainly focused on improving classification accuracy, we propose a model that can simultaneously assess accuracy, prediction confidence, and interpretability in wafer defect classification. To solve the class imbalance problem, we used a weighted cross-entropy loss function and convolutional neural network–based model to achieve a high accuracy of 97.8% on the test dataset and applied a temperature-scaling technique to enhance confidence. Furthermore, by simultaneously employing local interpretable model-agnostic explanations and gradient-weighted class activation mapping, the rationale for the predictions of the model was visualized, allowing users to understand the decision-making process of the model from various perspectives. This research can provide a direction for the next generation of intelligent quality management systems by enhancing the applicability of the proposed model in actual semiconductor production sites through explainable predictions.

## 1. Introduction

In the modern world, the demand for semiconductors and their sophistication are rapidly increasing with the development of artificial intelligence (AI), the Internet of Things, and autonomous driving technologies. Semiconductor industries play an important role as a driving force for national economies [1]. The semiconductor manufacturing process is a complex integration of eight major processes that are completed through hundreds of steps for each wafer. Defects occurring in such complex processes directly result in lower product yield, thereby exerting a substantial impact on corporate profitability [2]. Among them, electric die sorting inspection is a process that electrically verifies whether the integrated circuit chips on a wafer are properly functioning. The results of this inspection are recorded in the form of a wafer bin map (WBM), enabling the visualization of defect patterns based on the presence and position of defects on each chip.

As the transition to smart factories accelerates, wafer quality management systems must evolve beyond traditional inspection concepts to real-time decision-making support systems. In current industrial practice, engineers visually inspect WBMs and classify patterns, a process that is time-consuming, costly, and prone to subjectivity. Therefore, there is a growing interest in applying AI techniques to the analysis of these processes to supplement the judgment of engineers. As a result, there has been recent interest in applying AI techniques to supplement engineers’ judgment [3,4].

Smart factories in the Industry 4.0 era necessitate trustworthy AI systems that enable real-time decision-making. Unlike previous research that has primarily emphasized improving classification accuracy, real-world manufacturing settings demand answers to questions regarding confidence in the model and the rationale behind its decisions.

In this paper, we propose a wafer defect classification system that integrates prediction confidence quantification and dual explainability to satisfy the practical needs of these industrial sites. In particular, it utilizes a convolutional neural network (CNN) architecture that can solve the problem of data imbalance and effectively reflect the characteristics of defect patterns, and realize risk-based quality control by measuring uncertainty through temperature scaling, enabling effective collaboration with domain experts with an explanation framework that combines local interpretable model-agnostic explanations (LIME) and gradient-weighted class activation mapping (Grad-CAM).

To address the issues of developing a reliable wafer defect classification system in a smart factory environment, this paper is structured as follows. In Section 2, we describe a CNN-based classification model and Grad-CAM visualization technique for developing real-time decision-making support systems and examine the enhancements in processing speed and consistency relative to conventional manual inspection approaches. In Section 3, we introduce a temperature-scaling-based approach for quantifying predictive confidence to facilitate risk-based decision-making in industrial environments and detail a quality control decision-making process that leverages uncertainty information. In Section 4, we introduce a LIME-based explainability framework aimed at optimizing collaboration among domain experts and propose strategies to increase field operator confidence in AI systems. In Section 5, we assess the performance of wafer defect classification models, analyze the effectiveness of calibration techniques in enhancing predictive confidence, and report the visualization results of the model decision-making rationale using LIME and Grad-CAM.

## 2. Automated Wafer Defect Classification Model and Grad-CAM

### 2.1. WM-811K Dataset and Overall Architecture

A CNN is a deep neural network architecture that can automatically learn features from image data. Unlike conventional methods, it can effectively recognize visual patterns such as the shape, size, and position of defects without requiring manual feature extraction. In particular, it is suitable for processing defect data in the form of two-dimensional images, such as wafer maps, and can distinguish subtle differences in defect patterns through filter (kernel) operations that consider the local spatial structure. In addition, polling and activation operations can be used to learn abstract, high-dimensional features in a stepwise fashion, while achieving computational efficiency and generalization performance. These structural properties of CNNs make them a powerful tool for accurate classification and detection of different types of wafer defects [5,6].

Therefore, herein, we consider wafer map data as images and apply a CNN to automatically classify defective wafers. For each wafer map, the model is designed to automatically classify the wafer as good or defective using the normality assessment results as the ground truth for training. CNNs not only excel at automatically extracting meaningful features from images but also have the advantage of being able to learn in manufacturing environments with ongoing data accumulation, thus rendering them particularly suitable for industrial applications [7].

**Figure 1:**
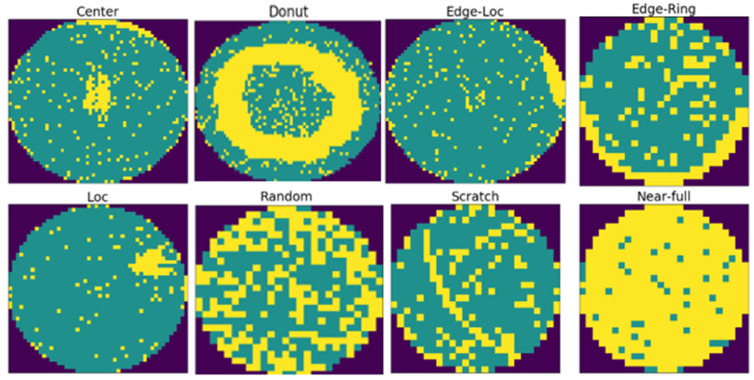
Classification of defect types in the WM-811K dataset.

In this study, we used a public dataset, WM-811K, which contains 811,456 wafer images and defect labels from real semiconductor processes, making it extremely useful for model training and generalization performance evaluation. Figure 1 shows an image of a real wafer from this dataset.

**Figure 2:**
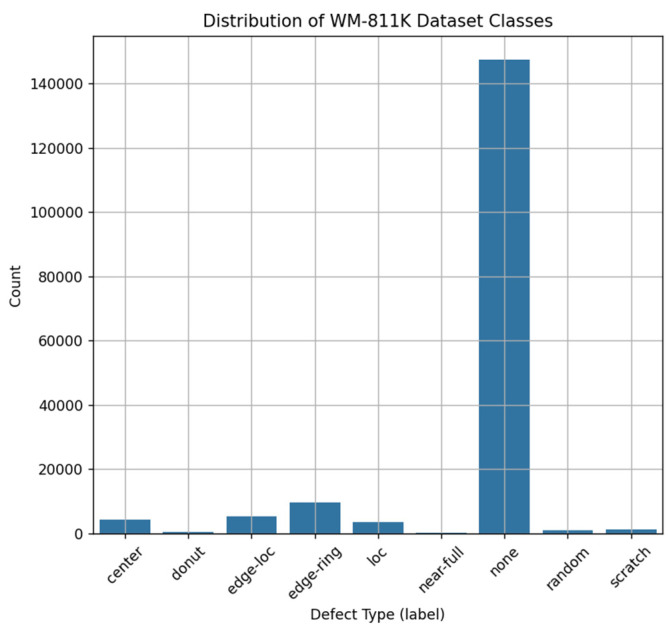
Distribution of WM-811K dataset classes.

However, as shown in Figure 2, this dataset is characterized by a strong imbalance between classes, with a large number of None (no defects) classes (over 140,000). In this study, we applied strategies such as class-specific oversampling and data augmentation to address this data imbalance problem [8]. The preprocessing of wafer defect images involved normalization, resizing, and conversion to grayscale. Moreover, techniques such as batch normalization and dropout were applied to facilitate efficient learning and achieve high accuracy.

**Figure 3:**
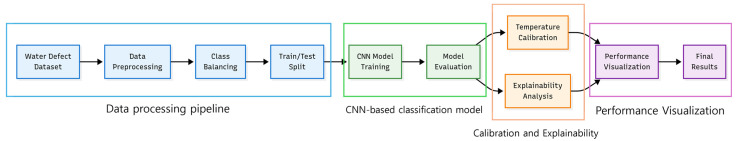
Overall system architecture for wafer defect classification.

Figure 3 shows the overall structure of the proposed wafer defect classification system, which consists of four main components. First, the data processing pipeline (blue) sequentially performs preprocessing of the WM-811K wafer defect dataset, class balancing, training/test splitting, and Pytorch (v2.8.0)-based dataset class construction. Second, the CNN-based classification model (green) is a key component that considers wafer map images as input and classifies defect types, including the process of model definition, training, and evaluation. Third, the confidence quantification module (yellow) applies the temperature-scaling technique through the calibration module to calibrate the prediction probability, calculate the expected calibration error (ECE)/maximum calibration error (MCE) metrics, and generate the reliability diagram. Fourth, the explainability analysis module (yellow) offers two approaches. The local interpretation based on LIME and the global interpretation using Grad-CAM provide multiple visualizations of the predictive rationale of the model.

The results from these two analysis modules are combined in the performance visualization (purple) and output as final results. This unified structure allows the realization of a practical system that simultaneously achieves high classification performance, predictive confidence, and interpretability.

### 2.2. Data Preprocessing and Class Balancing

The WM-811K dataset has issues with missing values and class imbalance. To address this, we first removed the missing values and split the data into training and test sets using Train_test_split. In this study, we defined the task as a multiclass classification problem for six representative defect classes.

To solve the class imbalance problem, we adopted a loss function-level approach instead of data augmentation techniques. The weighted cross-entropy loss function used in typical multiclass classification problems assumes that each class has an equal number of samples. Therefore, imbalanced datasets can lead to degraded learning performance for minority classes [9], consistent with prior CNN-based studies on wafer map defect classification under an imbalanced setting [10].

To address this issue, we weighted the cross-entropy loss function according to the number of samples per class. The weight $w_{i}$ for each class i is automatically calculated via scikit-learn’s compute_class_weight function, and the overall loss is defined as follows:

(1) $$L_{CE}\left(x,y\right)=-\sum_{i=1}^{C}w_{i}\cdot y_{i}\cdot \log\left(p_{i}\right)$$

where C is the number of classes, $y_{i}$ is the one-hot encoding vector of correct labels, and $p_{i}$ is the prediction probability for class i calculated via the Softmax function. At this step, the weight $w_{i}$ is set to be inversely proportional to the frequency of occurrence of class i in the entire dataset to prevent the underrepresentation of the learning loss of minority classes. This weighted cross-entropy loss function approach can effectively improve learning performance for minority classes while maintaining the natural distribution of the original data. Additionally, it offers the advantage of enabling more stable learning by avoiding artificial sample generation and overfitting risks associated with data augmentation [11,12].

### 2.3. Implementation of the CNN-Based Classification Model

**Figure 4:**
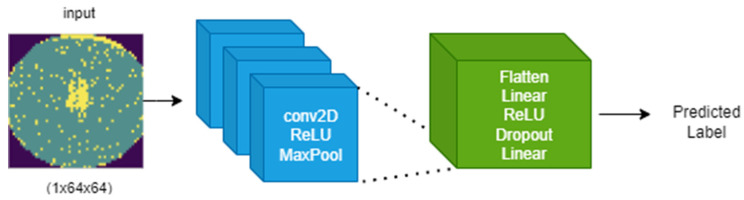
CNN Architecture.

The proposed CNN model follows the architecture illustrated in Figure 4, comprising three iterations of the Conv2D → ReLU → MaxPool block, followed by a sequence of Flatten → Linear → ReLU → Dropout → Linear.

The input image has a size of 64 × 64, and the number of channels is 1. The network has a shallow depth but was designed to effectively extract spatial features by having a sufficient number of filters at each step [13]. The first convolutional layer uses 32 filters, followed by 64 and 128 filters in the second and third layers, respectively, so that increasingly complex spatial features can be learned. After each convolutional layer, the ReLU activation function and MaxPooling operation are applied together to simultaneously perform feature extraction and dimensionality reduction.

In particular, we applied a dropout (*p* = 0.5) before the final liner layer to prevent overfitting. The number of neurons in the output layer is equal to the number of classes (6), and multiclass classification is performed using weighted cross-entropy loss function and Softmax. Our model is designed for deployment on real-time wafer inspection equipment. This requires fast inference speeds capable of processing hundreds of images per second and a low memory footprint, which were essential goals for our project. Although existing models offer high performance, they are too computationally heavy to meet these requirements. For this reason, we designed a lightweight, custom architecture to address these specific constraints. Recent studies have also explored ensemble learning approaches for wafer defect classification, demonstrating improved robustness and accuracy in semiconductor manufacturing contexts [14].

### 2.4. Implementation of the Grad-CAM-Based Visual Explanation Module

The Grad-CAM technique was used to provide a visual explanation of the predictive rationale of the model. This technique uses the output of the final convolutional layer of the CNN and the gradient of the predicted class to visually highlight the regions in the input image that influenced the classification decision [15].

In our Grad-CAM implementation, we set a specific convolutional layer of the model (model.net [3]) as the target layer and generated CAM images for each of the samples with correct and incorrect predictions. This enabled us to intuitively identify the regions on which the decisions of the model were based. For example, if the predicted label and true label matched, the model tended to focus on the center of the defect, whereas if the labels did not match, it tended to focus on the surrounding noise or the edges.

**Figure 5:**
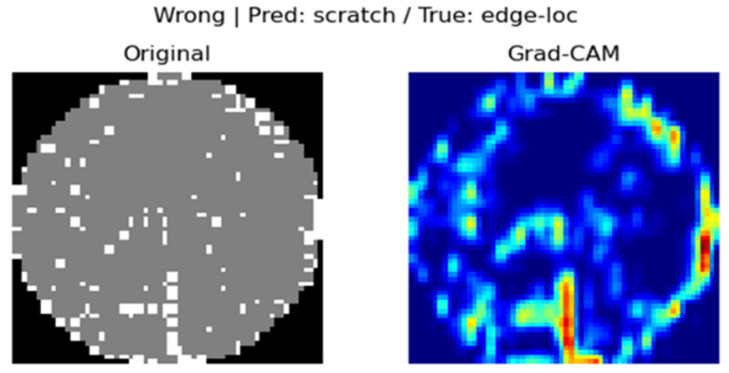
Results of Grad-CAM incorrect prediction.

Figure 5 displays a visualization of the prediction basis of the wafer map classification model using the Grad-CAM technique. The image on the left is the original wafer map, and that on the right is the Grad-CAM result, highlighting the regions that contributed the most to the classification decision of the model. The regions colored in red represent the spatial features that the model responded to most strongly in the prediction and are used to determine whether they are representative of the actual defect. In this outcome, we can visually see that the model predicted the “scratch” class but the actual class was “edge-loc”, indicating that the bottom pattern that Grad-CAM focused on led to a misclassification.

### 2.5. Training Configuration and Performance Evaluation

The training lasted for 25 epochs and used the Adam optimizer (LR = 0.001, Weight_decay = 1 × 10^−4^). The training/validation loss and accuracy were visualized at each epoch and ultimately achieved an accuracy of more than 98%. The final model performance was evaluated using a separately partitioned test dataset, and accuracy and confusion matrix as the evaluation metrics. The confusion matrix offers a visual representation of interclass misclassifications. For classes with comparatively lower performance, the predictive rationale of the model was analyzed through combined Grad-CAM and LIME visualizations. Our evaluation of the model performance distinguishes itself from previous studies by assessing accuracy and interpretability derived from the confusion matrix and effectiveness of explainable visualization techniques [16,17].

### 2.6. Comparative Analysis with State-of-the-Art Architectures

To validate the effectiveness of our proposed CNN architecture and address potential concerns regarding the use of more sophisticated pre-trained models, we conducted comprehensive comparative experiments with three widely-adopted state-of-the-art architectures: ResNet, EfficientNet, and MobileNet. All models were evaluated under identical experimental conditions to ensure fair comparison.

As summarized in Table 1, the proposed CNN architecture achieved an accuracy of 98.7%, which is comparable to or even surpasses that of deeper pre-trained models such as ResNet (98.91%) and EfficientNet (98.15%). Notably, our CNN attains this level of performance with only 2.19 M parameters, which is significantly fewer than ResNet (11.2 M) and EfficientNet (5.3 M). Furthermore, the proposed model delivers the fastest inference time (0.067 ms per image), highlighting its suitability for real-time wafer inspection applications. These results collectively validate the effectiveness and practicality of the proposed CNN, striking a favorable balance between accuracy, computational cost, and deployment feasibility.

Deep-learning-based wafer defect classification models have demonstrated high accuracy. However, the probability values output by the models often do not match the actual probability of a correct answer. These discrepancies can reduce the confidence of the model and cause risks in practical applications. Alternatively, if the confidence of the model in predicting probabilities is low, there is a risk of overlooking serious defects owing to incorrect predictions that the model is confident about. Therefore, increasing the confidence of the model is imperative. Recent studies, such as Adaptive Temperature Scaling (ATS), have been proposed to address limitations of the standard approach, particularly under class imbalance and out-of-distribution scenarios [18].

Despite its relatively simple implementation, temperature scaling is recognized as a highly effective calibration technique for improving the confidence of prediction probabilities [19]. Hence, in this study, we used it for enhancing the confidence of the CNN-based models trained for semiconductor wafer defect classification.

Temperature scaling is a straightforward and effective post hoc calibration method that modulates the Softmax probability distribution by scaling the output logits of the model with the temperature parameter (*T*). The technique can improve the probability output of existing models without retraining and has shown consistent performance improvements in numerous domains, making it a practical calibration technique. A typical Softmax can be obtained by applying a temperature parameter T as follows:

(2) $$\hat{p_{i}}=\frac{\exp\left(z_{i}/T\right)}{\sum_{j}\exp\left(z_{j}/T\right)}$$

where $\hat{p_{i}}$ is the Softmax probability output calibrated for class i and $z_{i}$ is the logit value for class i, i.e., the real value that the model outputs at the final layer. In addition, $\sum_{j}\exp\left(z_{j}/T\right)$ is the exponential sum of the temperature-adjusted logits for the entire class, corresponding to the denominator used to normalize the calibrated Softmax values. The temperature parameter *T* controls the shape of the distribution of the Softmax function. If T = 1, it operates identically to the normal Softmax. If T > 1, the output probability distribution becomes more uniform, reducing overconfidence of the model. Conversely, if T < 1, the probability distribution becomes sharper, producing predictions with higher confidence. However, it is generally not used, as it can lead to overconfidence.

### 2.7. Expected Calibration Error and Maximum Calibration Error

The calibration performance of the model was evaluated using the following two metrics. First, the expected calibration error (ECE) divides the overall prediction into several confidence intervals and calculates a weighted average of the difference between the average confidence and the actual accuracy of the model in each interval. The formula is as follows:

(3) $$ECE=\sum_{m=1}^{M}\frac{\left|B_{m}\right|}{n}\left|acc\left(B_{m}\right)\;-\;conf\left(B_{m}\right)\right|$$

where M represents the number of confidence intervals or bins. $B_{m}$ is the set of prediction samples that belong to the m th confidence bin. $\left|B_{m}\right|$ represents the number of samples contained in that mth bin. n is the total number of prediction samples. Moreover, $acc\left(B_{m}\right)$ represents the actual accuracy of the samples in bin $B_{m}$ and $conf\left(B_{m}\right)$ represents the average confidence of the samples in that bin.

ECE provides an overall indication of how overconfident or underconfident the prediction is, with values closer to 0 being closer to the ideal calibration state. For example, a prediction with a confidence of 90% and an actual accuracy of 70% would result in a calibration error of 20% in that bin. ECE is the average of these errors across all intervals. If ECE is a measure of the average quality of a calibration, the maximum calibration error (MCE) across all confidence bins represents the worst case. The MCE is calculated as follows:

(4) $$MCE=\max m\in \left\{1,\ldots ,M\right\}\left|acc\left(B_{m}\right)\;-\;conf\left(B_{m}\right)\right|$$

The MCE represents the largest value of the difference between the accuracy $acc\left(B_{m}\right)$ and average confidence $conf\left(B_{m}\right)$ in all confidence bins $B_{m}$. Alternatively, it serves as a metric that indicates the greatest degree of discrepancy in the model predictions. A high MCE value arises when the model exhibits extreme overconfidence or underconfidence in at least one interval [20,21].

### 2.8. Analysis of the Effects Before and After Calibration

**Figure 6:**
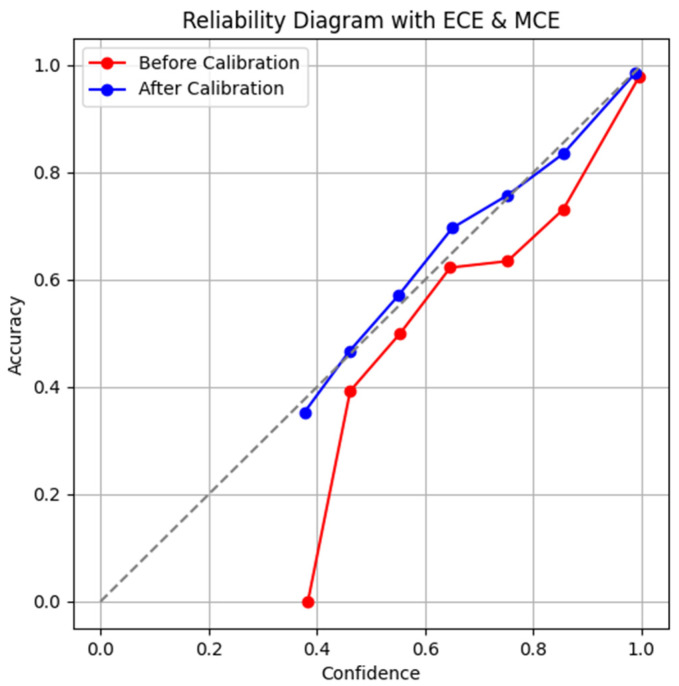
Reliability diagram with ECE and MCE.

To analyze the effects of applying the calibration technique, the reliability diagram visualizes the difference between the model prediction confidence and actual accuracy before and after applying the temperature scaling. Figure 6 is a reliability diagram showing the relationship between confidence and accuracy before and after calibration. The x-axis represents the confidence predicted by the model, and the y-axis represents the actual accuracy in that confidence interval.

ECEs and MCEs can be intuitively interpreted through the reliability diagram. A perfectly calibrated model lies on the diagonal (y = x), and the degree of deviation from this diagonal is reflected in ECE and MCE. A graph below the diagonal indicates that the model is overconfident, whereas that above the diagonal indicates that the model is underconfident. Before calibration (red line), there are some bins where the confidence value is high; however, the actual accuracy is relatively low, indicating that the model is overconfident. For example, we can see that a bin predicted with a probability of 0.6 has an actual accuracy of only ~0.4, which corresponds to an error of over 20%. By contrast, after calibration (blue line), we can see that most of the confidence bins are close to the diagonal and the probability output of the model has improved to a reliable state that matches the correct answer distribution. These results reveal that the temperature-scaling technique is an effective calibration method

## 3. Model Interpretation Using LIME

### 3.1. LIME Overview

Deep-learning-based classification models can achieve high accuracy, but the complexity of the training process and the opacity of the internal architecture of the model make it challenging to clarify the reasoning behind certain choices. This black-box problem is a major limitation in industrial environments where safety and reliability are critical, such as in real smart factories. LIME is a technique for providing interpretable explanations for individual predictions, regardless of the model architecture. LIME generates samples locally around the input data and trains a simple surrogate model (typically, a linear regression model) based on the predictions for these samples. It then provides coefficients indicating the contribution of each input feature to the prediction [22].

In this study, we used LIME to visually interpret the impact of each pixel in the wafer map image on the defect classification results. This allows for an intuitive interpretation related to the recognition of defect patterns and positions by the model. LIME derives an explanatory model through the following optimization process:

(5) $$explanation\;\left(x\right)=\arg\underset{g\in G}{\min}L\left(f,g,\pi_{x}\right)\;+\;\Omega \left(g\right)$$

This equation represents the process of finding a surrogate model g within the function space to describe the original complex model. Here, argmin (argument of the minimum) is the input value (variable) that minimizes the function. $L\left(f,g,\pi ₓ\right)$ denotes the local fidelity loss between the original model f and the surrogate model g. π_x_ is a distance-based weighting function defined for samples around the input *x* emphasizing the approximation accuracy in the neighborhood of *x*. In addition, Ω(g) is a regularization term that controls the complexity of the surrogate model, preventing overly complex explanations and ensuring the model simplicity and intuitiveness. Thus, this equation can be viewed as an optimization problem to find a surrogate model g near input *x* that approximates the original model well but is simple and easy to describe and understand [23]. The local fidelity loss function used by LIME to approximate the complex original model is defined as follows:

(6) $$L\left(f,g,\pi_{x}\right)=\sum_{z,z^{\prime }\in Z}\pi_{x}\left(z\right){\left(f\left(z\right)\;-\;g\left(z^{\prime }\right)\right)}^{2}$$

where f(z) is the value predicted by the original model for the input z and $g\left(z^{\prime }\right)$ is the value predicted by the surrogate model for the input $z^{\prime }$. The function $\pi_{x}\left(z\right)$ is a distance-based weighting function between the inputs *x* and *z*, where data closer to *x* is weighted heavily to increase the accuracy of the approximation in that region. The term ${\left(f\left(z\right)-g\left(z^{\prime }\right)\right)}^{2}$ represents the squared error between the predictions of the original and those of the surrogate model. Thus, this equation formulates an optimization objective that leads the surrogate model *g* to approximate the original model f as closely as possible for the local data around the input *x*. This is a key principle that enables the local explainability that LIME aims to achieve.

### 3.2. LIME Overview

To apply LIME, we selected some sample images from our test dataset. Each wafer map image comprises two-dimensional grayscale data. However, since the LIME library typically requires a 3-channel (RGB) input, the grayscale (1-channel) wafer map images were converted to the RGB format by replicating the single-channel data three times. In this study, we used the simple linear iterative clustering technique to divide the wafer map into a number of super pixels. We then generated several combinations of images with each super pixel disabled or retained, fed them into the CNN model, and observed the resulting prediction probabilities. Based on these predictions, LIME trains a linear regression-based surrogate model, weighted by the contribution of each super pixel to the prediction. These weights are visually masked or outlined to allow intuitive interpretation by the user.

### 3.3. Results and Interpretation

**Figure 7:**
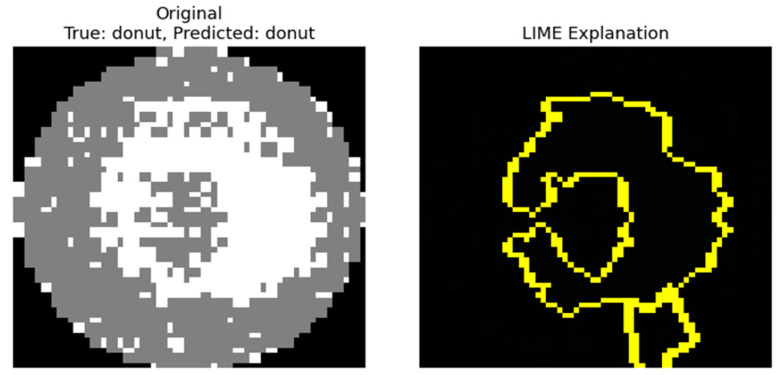
Visual explanation of wafer defect classification using LIME.

Figure 7 visualizes the results of LIME predictions of defects on the wafer map. On the left is the original wafer map image, and on the right is the result, with the yellow outline highlighting the regions where LIME made a remarkable contribution to the prediction. The contours represent the super pixel units that the model recognized as most influential for determining the defect class.

The results of the analysis using LIME enable the following analysis. The model utilized the central regions of defect concentration and regions exhibiting distinct anomalous patterns as critical predictive rationale, which closely corresponded to the actual defect positions and geometries. On the contrary, the normal regions on the periphery of the wafer were excluded from the LIME visualization, indicating that they had little impact on the model predictions. These results demonstrate that the LIME technique provides an effective visualization of the model decision process. In particular, the regions of the image that contributed to the prediction are clearly delineated at the super-pixel level, allowing an intuitive understanding of which parts the model relied on to determine the defects. Therefore, visual descriptions using LIME can serve as a tool to improve the reliability of the model, and it may be useful in the future to help manufacturing sites review predictions or decide whether to perform additional inspections based on specific defect patterns [24].

### 3.4. Complementary Use of LIME and Grad-CAM

Although LIME and Grad-CAM are popular explanatory techniques for interpreting the predictions of deep learning models, they have distinct differences in the way they work and the focus of their interpretation. Grad-CAM uses gradient information from specific convolutional layers of the CNN to visualize the spatial regions of the input image that the model is focusing on. It provides a description that reflects high-level features based on the internal hierarchy of the model. LIME, by contrast, focuses on the relationship between inputs and outputs, constructing a local linear model based on the distribution of data around the inputs. This has the advantage of providing an intuitive interpretation of the importance of the input regions that contributed to the model predictions. In particular, LIME works in a model-agnostic way, and therefore it is applicable to classification models with different architectures.

This study exploits the complementary characteristics of the two interpretability methods to maximize the interpretability of the wafer map defect classification model. Grad-CAM reflects the hierarchical learning process within the model and highlights the regions of high-dimensional feature extraction that form the basis of its predictions, whereas LIME provides a concrete visualization of the low-dimensional input patterns in the image that influenced the model decision. Using LIME and Grad-CAM in parallel, we could analyze and validate the logic behind the predictions from multiple angles, which is challenging to capture with a single analysis technique. Using these two techniques in parallel can make a substantial contribution to the reliability and transparency of predictions in manufacturing environments.

### 3.5. Synergistic Integration of Grad-CAM and LIME for Model Interpretability

Grad-CAM provides a global perspective of the model’s decision process, while LIME explains predictions from a local perspective by analyzing super-pixel units. In this study, we demonstrate that combining these two techniques enhances the reliability of model predictions, which is difficult to assess using only a single method. We define the consensus score as a weighted sum that takes into account both the spatial overlap of important regions and the correlation of contribution distributions:

(7) A=α⋅IoU+β⋅r

where IoU represents the intersection ratio between Grad-CAM active regions and LIME positive super-pixel masks, and *r* is the correlation coefficient of contribution distributions between the two explanations. In this study, spatial agreement was considered a more important factor, so we set *α* = 0.7 and *β* = 0.3.

**Figure 8:**
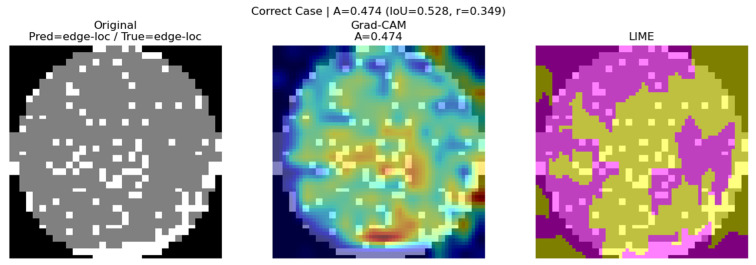
Correct classification case (Pred: edge loc/True: edge loc).

According to the metrics in Table 2, correct classifications with high consensus scores show relatively high IoU (0.528) and correlation coefficient (0.349), indicating that Grad-CAM and LIME consistently highlight overlapping regions. This consistency can be observed in Figure 8.

**Figure 9:**
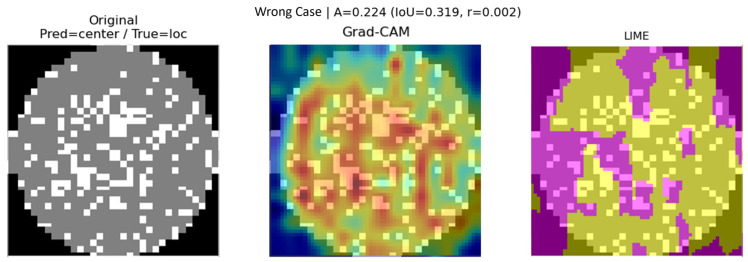
Wrong classification case (Pred: center/True: loc).

In wrong classification cases, the IoU (0.319) is partially maintained, but the correlation coefficient (0.002) is extremely low, suggesting that the two methods emphasize different patterns. This implies that the model focused on certain regions but derived its decision from insufficient or biased evidence, as illustrated in Figure 9.

**Figure 10:**
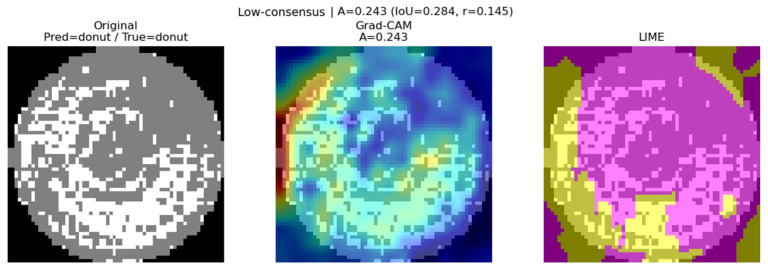
Low Consensus Correct classification case (Pred: donut/True: donut).

For correct classifications with low consensus, both IoU (0.284) and correlation coefficient (0.145) remain low. In such cases, the model predicts the correct label, but the regions highlighted by Grad-CAM and LIME do not align, representing a “correct but unclear rationale” scenario. An example of this case is presented in Figure 10.

These results reveal aspects of model reliability that cannot be captured by accuracy alone, showing that consensus-based evaluation enables a more fine-grained assessment. These findings substantiate the claim that the joint use of LIME and Grad-CAM enhances reliability assessment of deep learning models. By capturing both hierarchical feature-level attention (Grad-CAM) and localized super-pixel contributions (LIME), the combined framework allows more comprehensive interpretability than either method alone. This complementary perspective is particularly valuable for safety-critical manufacturing applications, where reliable defect classification is essential.

## 4. Evaluation of Model Accuracy and Confidence Calibration Performance

### 4.1. Verification of the Effectiveness of the Proposed Method

To verify the individual effectiveness of the core techniques proposed in this study, we conducted an ablation study. The experiment compared three model configurations: (1) the basic CNN model (Baseline), (2) the baseline model with weighted cross-entropy loss function applied, and (3) our final proposed model, which adds temperature scaling. All experiments were performed under the same dataset and hyperparameter conditions, and the results are shown in Table 3.

As seen in Table 3, applying the weighted cross-entropy loss function to the baseline model led to a significant increase in accuracy, from 93.93% to 97.8%. This demonstrates that the weighted cross-entropy loss function effectively mitigates the severe class imbalance issue in the WM-811K dataset, improving prediction performance for minority classes. Furthermore, while the accuracy remained unchanged after applying temperature scaling, the ECE, a metric for model confidence, dramatically decreased from 0.073 to 0.021. This indicates that temperature scaling successfully corrected the model’s overconfidence tendency, thereby enhancing prediction reliability while maintaining prediction accuracy. Therefore, this ablation study confirms that the two proposed techniques contribute independently to improving classification performance and reliability, respectively.

### 4.2. Performance Evaluation Metrics

**Figure 11:**
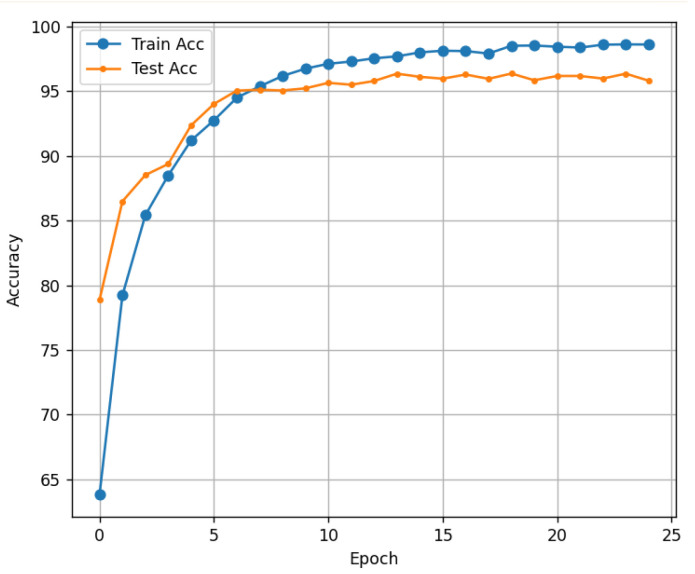
Result of accuracy graph.

In addition to accuracy, the performance evaluation utilized confusion matrix to quantitatively analyze the misclassification across classes. High accuracy was achieved for most classes, and underprediction in the source classes was minimized owing to the effect of the weighted cross-entropy loss function. Figure 11 shows the evolution of train accuracy and test accuracy per epoch during the training process. This graph shows that the model performance gradually improves as the number of epochs increases. The model was trained for 25 epochs, achieving training and test accuracies of 98.6% and 97.8%, respectively, demonstrating strong performance.

**Figure 12:**
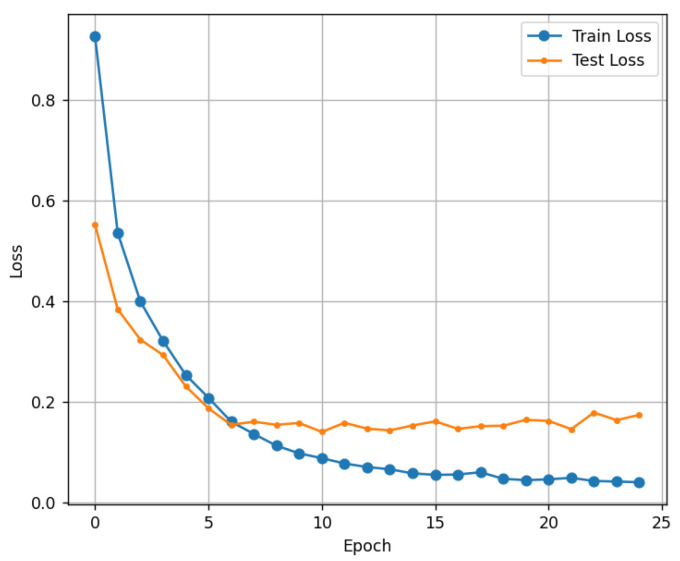
Result of loss graph.

Figure 12 illustrates the early stopping technique, which was implemented to mitigate the risk of overfitting and enhance the model’s generalization capability. Training was continuously monitored using the validation loss. If the validation loss did not improve for a specified number of consecutive epochs (e.g., a patience of 10 epochs), the training was automatically halted. This ensures that the final model selected is the one that exhibits optimal performance on unseen data, rather than merely memorizing the training set.

**Figure 13:**
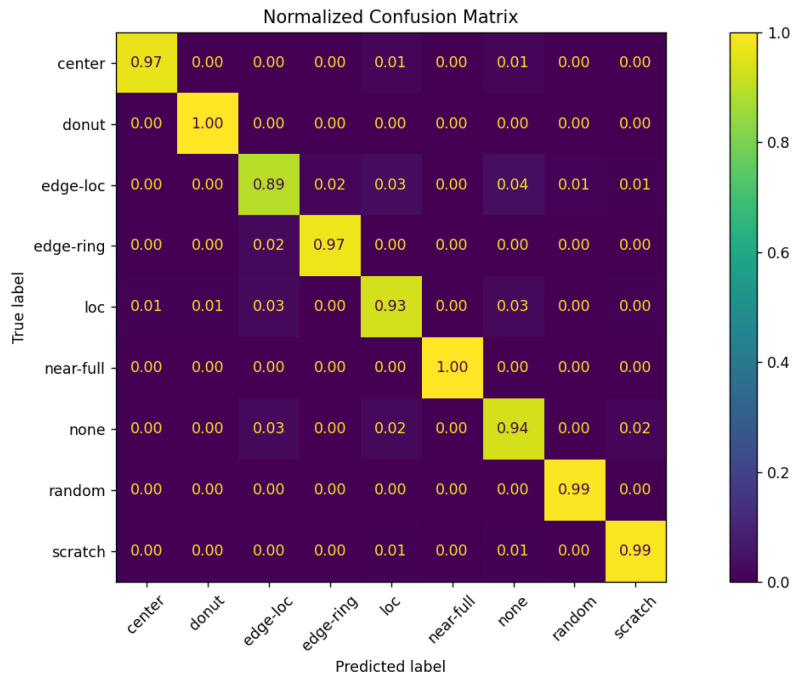
Normalized Matrix.

Figure 13 presents a normalized confusion matrix showing the prediction proportions for each combination of true and predicted classes. The value at position (i, j) indicates the proportion of samples whose true class is i and that were predicted as class j. In other words, the closer the diagonal values are to 1.0, the better the classification performance for the corresponding class.

### 4.3. Confidence Calibration Results

Temperature scaling, which was used as a calibration technique, mitigated the model overconfidence. Before calibration, some confidence bins showed deviations of more than 20% between the prediction probability and the actual accuracy, which was a major source of lower confidence.

Table 4 quantitatively shows that the calibration technique considerably improved the prediction confidence of the model. The output probabilities of the model have been adjusted to closely match the actual probability of the correct answers. In Figure 6, we can see that ECE improved from 0.073 to 0.021 and MCE improved from 0.209 to 0.068.

### 4.4. Visual Interpretation Results (Grad-CAM and LIME)

In this study, Grad-CAM and LIME were used to visually analyze the rationale behind the model predictions. Grad-CAM visualized the main regions that the model focused on in the form of a heatmap, whereas LIME identified the input regions that contributed to the prediction at the super-pixel level. In the case of Grad-CAM, Figure 5 shows that misclassified samples were primarily focused on the Edge or Edge-Loc regions, which served as a basis for identifying directions for model improvement. For LIME, we can see in Figure 7 that the input super pixel regions, which considerably contributed to the prediction results, are outlined in yellow. Most of them correctly recognized the center of the defect concentration, suggesting that the model has superior local interpretability.

**Figure 14:**
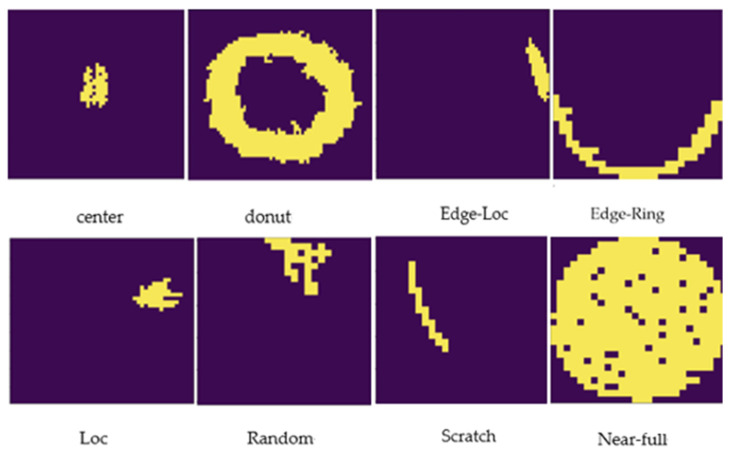
Visualization of wafer map image data.

Figure 14 selects images corresponding to a specific index and outputs them by defect type. For each image, the purpose is to highlight only the largest defect regions and remove others for visual clarity.

### 4.5. In-Depth Analysis of Misclassification Cases

**Figure 15:**
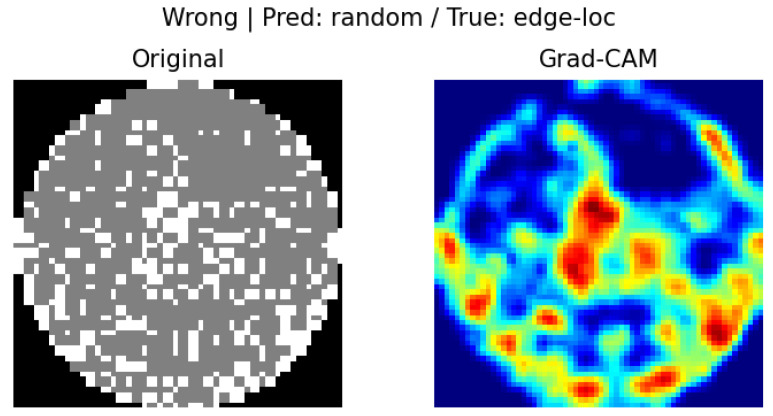
Image Misclassification Example Using Grad-CAM.

**Figure 16:**
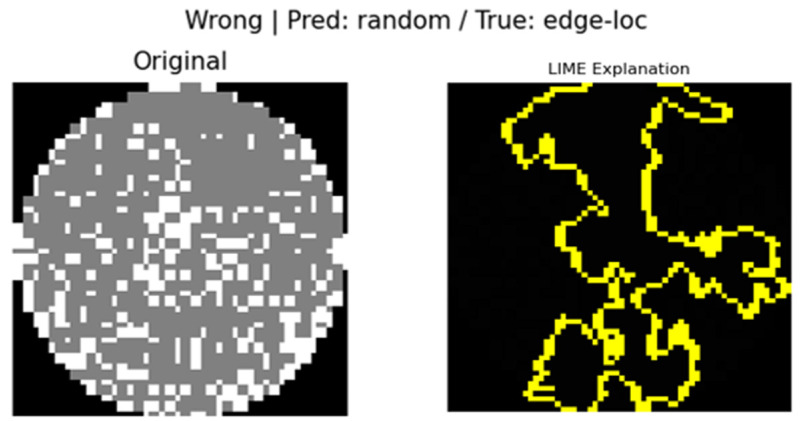
Image Misclassification Example Using LIME.

Figure 15 shows an image misclassification example using Grad-CAM, and Figure 16 presents the LIME analysis results, also supporting this point. The LIME visualization highlights that the area contributing to the model’s “random” prediction was an irregular pattern in the center of the image. This local feature is unrelated to the essential nature of an “Edge-loc” defect and proves that the model relied excessively on specific noise rather than recognizing the overall defect pattern. This visual analysis confirms that the main reason our model confuses “Edge-loc” and “Random” is due to similar visual features in the periphery, not the actual shape of the defects. This result suggests that future research needs a methodology for training the model to focus more on the unique features of defects to further improve its prediction reliability.

## 5. Conclusions

Previous research has focused on increasing the accuracy of defect classification through CNN-based architectures [25,26]. However, herein, we proposed an integrated system that simultaneously achieves high performance in wafer defect classification and predicts confidence and interpretability. This study incorporated the following three elements to enhance its practical value. First, a CNN-based model and weighted cross-entropy loss function were used to solve the class imbalance problem and achieve a high accuracy of 97.8%. Second, we quantified the prediction confidence by applying temperature scaling, which improved ECE by 71.2% and MCE by 67.5%, mitigating the problem of overconfidence in the model. Third, a dual explainability framework combining LIME and Grad-CAM was used to simultaneously visualize the prediction rationale of the model in input and feature spaces. This integrated approach has enabled the development of a system that goes beyond simple classification results, addressing practical questions such as “how confident is the model?” and “what is the basis for its judgment?” LIME provided a regional description in the input space, whereas Grad-CAM provided a feature-based interpretation inside the model, enabling a complementary understanding. Confidence quantification based on temperature scaling also allowed us to objectively determine whether a further review of uncertain predictions was necessary. The system provides comprehensive information, such as “This wafer is defect A (confidence: 87%), and the reason is the pattern in the center”, rather than simply stating, “This wafer is defect A”, greatly improving its applicability in practice. In future, it is expected to present a new paradigm for intelligent quality management systems by expanding it to various manufacturing processes and developing it into a real-time system.

## Figures and Tables

**Table 1:** Comparative performance of the proposed CNN architecture against state-of-the-art models (ResNet, EfficientNet, and MobileNet) on the WM-811K dataset.

Architecture	Accuracy (%)	Parameters (M)	Inference Time (ms)
CNN	98.7	2.192	0.067
ResNet	98.91	11.2	0.285
EfficientNet	98.15	5.3	0.120
MobileNet	95.01	3.216	0.156

**Table 2:** Comparison of quantitative metrics between correct and wrong classifications.

Metric	Correct Classification	Wrong Classification	Low Consensus
IoU	0.528	0.319	0.284
Correlation Coefficient	0.349	0.002	0.145
Agreement Score A	0.474	0.224	0.243

**Table 3:** Ablation Experiment Results of the Proposed Techniques.

Model Configuration	Accuracy	ECE
Baseline	93.93%	0.0358
Baseline + Weighted Loss	97.8%	0.073
Baseline + Weighted Loss + Temperature Scaling	97.8%	0.021

**Table 4:** ECE and MCE results before and after calibration.

Metric	Before Calibration	After Calibration
ECE	0.073	0.021
MCE	0.209	0.068

## Data Availability

The original contributions presented in this study are included in the article. Further inquiries can be directed to the corresponding author.
